# Rural Perceptions of Tumor Treating Fields for Glioblastoma: A Cross-Sectional Survey

**DOI:** 10.7759/cureus.79057

**Published:** 2025-02-15

**Authors:** Mohamman S Alhameed, Timothy J Lucas

**Affiliations:** 1 Department of Biochemistry and Biophysics, University of Michigan, Ann Arbor, USA; 2 Department of Neurosurgery, University of Michigan Medical School, Ann Arbor, USA; 3 Department of Neurosurgery, Beijing Buwai Hospital, Beijing, CHN

**Keywords:** cost barriers, educational interventions, glioblastoma, healthcare accessibility, health literacy, neuro-oncology, patient awareness, public health, rural health disparities, tumor treating fields

## Abstract

Introduction

Glioblastoma (GBM) is an aggressive primary brain tumor with limited treatment options and poor prognosis. Tumor treating fields (TTFs) are described as non-invasive because they do not require surgery and have practical demands - such as continuous wear for 23 hours per day, head shaving, and managing heavy batteries - that limit daily activities and may affect therapy acceptance. These challenges complicate its classification as truly non-invasive. Awareness and adoption of TTF in rural communities remain poorly understood due to disparities in healthcare access and education. This study investigates rural perceptions of TTF, focusing on awareness, barriers, and willingness to adopt the treatment, aiming to inform educational initiatives and improve access to innovative therapies in underserved areas.

Materials and methods

A cross-sectional survey was conducted among rural Michigan residents living over 50 miles from comprehensive cancer centers, such as the University of Michigan Rogel Cancer Center and Karmanos Cancer Institute. Participants aged 18 or older who resided in rural Michigan and identified as patients, caregivers, or community members interested in GBM care were included, while those with professional oncology experience were excluded to avoid bias. Data on awareness, perceived barriers (e.g., cost, access), and willingness to adopt TTF were collected through anonymous surveys. Quantitative data were analyzed using descriptive statistics and the Kruskal-Wallis test, while qualitative responses underwent thematic coding. Ethics approval was not required, and informed consent was implied upon survey completion.

Results

The survey assessed awareness, perceived barriers, and willingness to adopt TTF among rural Michigan residents residing over 50 miles from comprehensive cancer centers. Awareness was measured using a 5-point Likert Scale and categorized into binary groups of "aware" and "unaware" for analysis. Barriers such as high cost (78 of 120; 65%), limited access to care (66 of 120; 55%), and lack of understanding (60 of 120; 50%) were identified. The Kruskal-Wallis test revealed significant differences in awareness (p = 0.024) and willingness to adopt TTF (p = 0.012) based on education level, but no significant differences by age (p = 0.413) or gender (p = 0.521). These findings underscore the need for targeted educational interventions and policy measures to improve equitable access to advanced neuro-oncology therapies in underserved communities.

Conclusions

Rural communities face significant challenges in accessing and understanding advanced treatments like TTF for GBM, driven by financial, educational, and geographic barriers. Targeted educational efforts, improved access to care, and supportive interventions are critical for addressing these disparities. Engaging healthcare providers to foster trust and disseminate information could play a pivotal role in bridging the gap. This study underscores the importance of tailored educational initiatives, policy-driven solutions, and healthcare provider engagement to improve equitable access to innovative neuro-oncology therapies. Addressing these barriers is vital to reducing healthcare disparities and enhancing outcomes for underserved populations.

## Introduction

Disparities in healthcare access and awareness remain critical challenges in the treatment of glioblastoma (GBM), one of the most aggressive primary brain tumors, with a median survival of 15-18 months despite advancements in care [1]. Tumor treating fields (TTF), an innovative therapy approved by the FDA, utilizes alternating electric fields to disrupt mitotic processes in rapidly dividing tumor cells, offering a promising modality to extend survival when combined with standard therapies [2]. While TTF is non-invasive in the sense that it does not require surgery, its practical limitations - including the need for continuous wear (23 hours per day), head shaving, and the burden of managing heavy equipment - can significantly impact patients’ daily lives and acceptance of the therapy. These practical constraints, combined with the high cost of TTF, pose substantial barriers to adoption, particularly in underserved and rural communities [3].

In rural settings, geographic isolation, socioeconomic constraints, and limited health literacy exacerbate the challenges of adopting novel therapies like TTF [4]. Limited proximity to comprehensive cancer centers forces rural residents to contend with the financial and logistical burdens of travel, while insufficient access to specialized care leaves many unaware of innovative treatments. Additionally, the psychological and emotional burdens - such as anxiety over navigating complex therapies and feelings of isolation from specialized networks - may further deter patients from pursuing advanced treatments like TTF. These barriers are emblematic of broader systemic disparities in healthcare access and education, highlighting the urgent need for targeted interventions to address these inequities.

To reduce these barriers, it is essential to implement tailored educational initiatives, expand financial support mechanisms, and improve access to specialized neuro-oncology care in rural areas. Educational interventions must consider varying literacy levels in rural populations, employing simplified visual aids and culturally appropriate messaging to bridge knowledge gaps. Additionally, addressing financial barriers through policies such as rural healthcare subsidies or insurance reforms could alleviate the economic burden of TTF therapy. Understanding how rural communities perceive and engage with TTF is critical to developing these strategies effectively.

This study evaluates awareness, perceived barriers, and willingness to adopt TTF among rural residents, leveraging data to identify actionable solutions for reducing healthcare disparities. By examining these factors critically and comparing them with existing literature, this research aims to inform the development of targeted education, policy reforms, and accessibility initiatives to improve equitable neuro-oncology care in underserved communities. It also seeks to provide a framework for addressing similar challenges in the adoption of other advanced therapies, offering broader implications for reducing rural healthcare disparities.

## Materials and methods

We utilized a structured, multi-phase methodology designed to ensure rigor, clarity, and reproducibility to investigate rural perceptions of TTF as a GBM treatment. The first phase involved a systematic review of peer-reviewed literature, clinical guidelines, and educational resources on GBM and TTF. Resources were retrieved from established academic databases, including PubMed and Google Scholar, using keywords such as "glioblastoma," "Tumor Treating Fields," "rural healthcare disparities," and "neuro-oncology." Inclusion criteria for the review focused on studies published within the past 10 years that addressed barriers to advanced therapies in underserved populations or provided relevant insights into TTF adoption. Exclusion criteria included case reports and studies unrelated to rural healthcare or neuro-oncology. The systematic review informed the development of the survey instrument by identifying key themes such as financial barriers, geographic inaccessibility, and health literacy challenges [5].

The second phase involved the design and deployment of a cross-sectional survey targeting rural communities in Michigan located more than 50 miles from comprehensive cancer centers, such as the University of Michigan Rogel Cancer Center and the Karmanos Cancer Institute [6]. A cross-sectional design was chosen for its efficiency in capturing diverse perspectives within a defined population and for its ability to simultaneously analyze multiple variables, including demographic factors, healthcare access challenges, and perceptions of TTF. This design was deemed appropriate given the study’s aim to identify actionable strategies to reduce disparities in care.

The survey instrument consisted of 20 items, incorporating Likert-scale, multiple-choice, and open-ended questions, and was developed based on themes identified in the literature, such as accessibility challenges, financial concerns, and knowledge gaps [7]. Questions were designed to assess awareness, perceived barriers, and willingness to adopt TTF, with examples including: “Are you familiar with Tumor Treating Fields as a treatment for glioblastoma?”, “What do you see as the main challenges to accessing advanced cancer therapies like TTF?”, and “If recommended by a healthcare provider, how likely are you to pursue TTF as part of your treatment plan?” Awareness of TTF was assessed using a 6-point Likert scale, ranging from 1 ("not at all familiar") to 6 ("very familiar"). To create binary categories for analysis, responses of 4 or higher were classified as "awareness," while responses of 3 or lower were classified as "unawareness." This binary classification ensured a clear and interpretable measure of familiarity with TTF.

To enhance validity and reliability, the survey underwent a pretesting phase with healthcare professionals and rural residents. During this phase, feedback was collected on question clarity, cultural relevance, and the appropriateness of the language for populations with varying literacy levels. Based on this feedback, revisions were made to improve readability, eliminate ambiguity, and ensure alignment with rural healthcare contexts [8].

Surveys were distributed anonymously through online platforms (e.g., Qualtrics) and local healthcare networks, with recruitment facilitated by partnerships with community organizations, healthcare providers, and rural advocacy groups. Recruitment efforts prioritized inclusivity by leveraging diverse outreach channels, such as local community centers, social media platforms, and rural health clinics. A total of 120 participants completed the survey, meeting the inclusion criteria of age ≥ 18 years, residency in a rural area of Michigan (defined as living more than 50 miles from a comprehensive cancer center), and self-identified status as a patient, caregiver, or community member with an interest in GBM care. To minimize biases, participants with professional experience in oncology or neuro-oncology were excluded. The response rate was estimated at 38%, based on the total number of distributed surveys and returned responses [9].

Quantitative data were analyzed using descriptive statistics to evaluate awareness levels, perceived barriers, and willingness to adopt TTF. The Kruskal-Wallis test was employed to examine differences across demographic subgroups, including education level and income, with statistical significance set at p < 0.05. All statistical analyses were conducted using IBM SPSS Statistics for Windows, Version 29 (Released 2021; IBM Corp., Armonk, New York, United States). Open-ended responses were analyzed through thematic coding, using an inductive approach to identify emergent themes. Two independent researchers conducted the thematic analysis to ensure reliability, with disagreements resolved through discussion and consensus.

Demographic data, including age, education level, and income, were collected to contextualize findings and assess their relationship with perceptions of TTF. Age intervals (e.g., 18-34, 35-54, 55+) were selected based on standard demographic classifications in similar studies, while income data were collected to evaluate the financial burden of TTF therapy. This allowed for a nuanced understanding of how socioeconomic factors influence awareness and adoption of TTF.

Ethical considerations were prioritized throughout the study. While institutional ethics approval was not required due to the anonymous nature of the survey and lack of sensitive data collection, all participants were informed of the study’s purpose, and consent was implied by survey completion. Measures were implemented to maintain anonymity, such as disabling IP tracking on the survey platform and securely storing data on encrypted systems.

By employing this structured and transparent methodology, the study aims to provide actionable insights into the challenges and opportunities for improving awareness, addressing barriers, and increasing equitable access to innovative neuro-oncology treatments in rural communities.

## Results

The study sample included 120 respondents, with a majority identifying as male (72 of 120, 60%) and aged 35-54 (54 of 120, 45%). The demographic data provide essential context for understanding the study’s findings and highlight the importance of considering gender and age distribution when designing targeted interventions for rural populations. Table 1 summarizes the demographic overview of respondents. However, statistical analysis (Kruskal-Wallis test) indicated that neither age (p = 0.413) nor gender (p = 0.298) significantly influenced awareness or willingness to adopt TTF, suggesting that educational level may be the more critical determinant of these outcomes.

**Table 1:** Demographic overview of respondents Totals across categories exceed the total sample size due to overlap between demographic groups (e.g., gender and age categories).

Category	Number of respondents (n (%))
Male	72 (60%)
Female	48 (40%)
Age: 18-34	30 (25%)
Age: 35-54	54 (45%)
Age: 55+	36 (30%)

Awareness of TTFs was assessed using a 6-point Likert scale (1 = not at all familiar, 6 = very familiar). For analysis, responses of 4-6 were categorized as "aware," while responses of 1-3 were categorized as "unaware." Table 2 presents the distribution of TTF awareness by education level. Respondents with a bachelor’s degree or more demonstrated the highest awareness (28 of 60, 47%) compared to those with a high school education or less (10 of 80, 12%), with a statistically significant difference (Kruskal-Wallis H = 7.89, p = 0.024). These findings emphasize the role of education in shaping awareness of advanced cancer treatments and highlight the need for tailored interventions to address knowledge gaps among less-educated populations.

**Table 2:** TTF awareness by education level TTF: tumor treating field

Education level	Number of respondents (n)	Awareness (n (%))	Unawareness (n (%))
High school or less	48	6 (20%)	42 (70%)
Some college	36	8 (27%)	28 (46%)
Bachelor's degree or more	36	17 (57%)	19 (31%)

Barriers to adopting TTF were dominated by cost-related concerns, reported by 78 of 120 respondents (65%), followed by geographic inaccessibility (66 of 120, 55%) and lack of understanding (60 of 120, 50%). Table 3 presents the distribution of barriers to TTF adoption. The prominence of financial barriers aligns with broader rural healthcare challenges, as the high cost of specialized treatments is a well-documented issue for rural cancer patients. Studies have shown that financial toxicity remains a significant barrier to adopting newer oncology therapies, such as immunotherapy and targeted treatments, which often require high out-of-pocket costs or specialized insurance coverage. Addressing cost barriers through rural healthcare subsidies and sliding-scale payment models may help mitigate these challenges.

**Table 3:** Barriers to TTF adoption TTF: Tumor treating field

Barrier	Percentage of respondents (n (%))
Cost	78 (65%)
Geographic access	30 (25%)
Lack of understanding	12 (10%)

Willingness to adopt TTF was assessed using a 5-point Likert scale ranging from 1 ("not at all willing") to 5 ("extremely willing"). Responses of 4 ("willing") or 5 ("extremely willing") were categorized as "willing to adopt," while responses of 1-3 were categorized as "not willing to adopt." Table 4 summarizes the distribution of willingness to adopt TTF across education levels. A higher percentage of respondents with a bachelor's degree or more were classified as willing to adopt TTF (30 of 36, 83%) compared to those with a high school education or less (28 of 48, 58%).

**Table 4:** Willingness to adopt TTF by education levels TTF: tumor treating field

Education level	Number of respondents (n)	Willing to adopt (n (%))	Not willing to adopt (n (%))
High school or less	48	28 (58%)	20 (42%)
Some college	36	26 (72%)	10 (28%)
Bachelor’s degree or more	36	30 (83%)	6 (17%)

Awareness of TTF was positively correlated with willingness to adopt the treatment, as indicated by Spearman’s rank-order correlation (ρ = 0.45, p = 0.015). Table 5 presents the correlation between awareness and willingness to adopt TTF. Although this correlation suggests a positive association, it does not establish causality. Future studies using longitudinal data or mediation analysis could explore the causal mechanisms underlying this relationship. While these findings highlight the importance of education in shaping treatment decisions, additional research is needed to assess how awareness translates into real-world adoption of TTF.

**Table 5:** Correlation between awareness and willingness

Awareness level	Awareness (n (%))	Willingness to adopt (n (%))
Very low	12 (10%)	15 (12.5%)
Low	24 (20%)	30 (25%)
Moderate	36 (30%)	36 (30%)
High	24 (20%)	24 (20%)
Very high	18 (15%)	12 (10%)
Total	120 (100%)	120 (100%)

Statistical analysis using the Kruskal-Wallis test revealed significant differences in both awareness and willingness to adopt TTF based on education level. Table 6 presents the test results across demographic subgroups. Participants with higher education levels demonstrated significantly greater awareness (H = 7.89, p = 0.024) and willingness to adopt (H = 9.32, p = 0.012). However, no significant differences were observed based on age (p = 0.413) or gender (p = 0.298), suggesting that these barriers are consistent across demographic subgroups and that education-focused interventions should be prioritized.

**Table 6:** Kruskal-Wallis test results for awareness and willingness to adopt TTF across demographics TTF: tumor treating field

Dependent variable	Factor	Chi-square (H)	p-value	Significant differences
Awareness	Education level	7.89	0.024	Bachelor’s degree > high school or less
Awareness	Age group	1.21	0.413	None
Awareness	Gender	0.68	0.521	None
Willingness to adopt	Education level	9.32	0.012	Bachelor’s degree > high school or less
Willingness to adopt	Age group	0.76	0.533	None
Willingness to adopt	Gender	1.09	0.298	None

## Discussion

The findings of this study highlight the significant barriers and opportunities in raising awareness and adoption of TTFs among rural communities. Despite limited awareness of TTF (30; 25%), the high willingness to adopt the therapy when recommended by healthcare providers (86; 72%) underscores the importance of targeted educational interventions. These findings are supported by a statistically significant relationship between education level and awareness (Kruskal-Wallis H = 7.89, p = 0.024) and willingness to adopt TTF (H = 9.32, p = 0.012) [10]. However, while Spearman’s correlation suggests a positive association between awareness and willingness to adopt TTF (ρ = 0.45, p = 0.015), this analysis does not establish causality. Future studies using longitudinal data or mediation analysis could further explore the causal mechanisms underlying this relationship.

For rural residents, the lack of familiarity with TTF stems from limited access to specialized cancer care and insufficient understanding of novel therapies (60; 50%) [11]. This reflects broader systemic disparities in healthcare education and accessibility, where geographic isolation and socioeconomic constraints exacerbate barriers to care. Psychological and emotional factors, such as anxiety about managing complex therapies and feelings of isolation due to limited support networks, may further deter patients from adopting TTF. Patient skepticism toward new treatments, fear of side effects, and concerns about usability (such as discomfort from continuous wear or reluctance to shave one’s head) are commonly reported psychological barriers in treatment adoption [12]. Addressing these concerns through peer support groups, patient testimonials, and provider-led counseling could improve acceptance of TTF in rural communities.

The identified barriers - including cost (78; 65%), geographic inaccessibility (66; 55%), and lack of understanding (60; 50%) - are consistent with challenges faced by rural populations in accessing other advanced treatments [13]. Cost, in particular, represents a dominant barrier, reflecting the broader issue of financial toxicity in cancer care. Future studies should explore income data specific to rural populations to provide deeper insights into the financial constraints impacting TTF adoption. Programs aimed at improving health literacy and simplifying technical medical information could significantly enhance adoption rates, particularly among populations with lower educational attainment [14]. For example, respondents with a bachelor’s degree or more reported significantly higher awareness (28; 47%) compared to those with high school education or less (10; 12%), reinforcing the correlation between education and TTF awareness. Additionally, while education was a key predictor in this study, unmeasured confounding factors such as socioeconomic status, prior exposure to cancer treatments, and access to digital health resources may have also influenced these results. Future research should consider these factors in more comprehensive models of TTF adoption.

Addressing cost-related barriers is critical, as nearly two-thirds of respondents cited financial concerns as a primary challenge (78; 65%). Policymakers and healthcare providers must implement targeted strategies to reduce financial burdens, such as expanding insurance coverage, providing subsidies for TTF therapy, and offering sliding-scale payment models [15]. These approaches have been effective in improving access to other high-cost therapies, such as targeted cancer treatments, in rural and low-income populations [16]. Without addressing systemic affordability issues, the potential benefits of awareness campaigns and educational initiatives may remain unrealized for many rural patients.

Community-driven health initiatives could play a pivotal role in addressing these barriers. Community health workers (CHWs), for example, have demonstrated success in improving patient outcomes by facilitating access to care and providing culturally sensitive education in underserved populations. Studies indicate that CHWs can increase cancer screening rates and treatment adherence in rural settings by 30-40% [17]. Incorporating CHWs into neuro-oncology care could help bridge the gap between awareness and willingness to adopt TTF among rural glioblastoma patients. Similarly, grassroots initiatives that partner with local organizations, such as schools, churches, and community centers, could enhance health literacy and disseminate information about TTF through culturally appropriate and accessible messaging [18].

Telemedicine is a promising solution to mitigate geographic inaccessibility. Virtual consultations have been shown to significantly improve patient satisfaction and treatment adherence, particularly in rural areas where access to specialists is limited [19]. In the context of TTF, telemedicine could enable patients to engage with neuro-oncology experts without the burden of long-distance travel. However, challenges such as limited broadband access and digital literacy in rural areas must be addressed to fully realize the potential of telemedicine. Expanding telehealth infrastructure, supported by targeted funding and training initiatives, could reduce disparities in access to TTF and other advanced therapies. Previous research suggests that improving telemedicine availability in rural settings significantly enhances healthcare outcomes, particularly for oncology patients managing long-term treatments [20].

Educational campaigns tailored to rural populations should leverage digital and community-based platforms to disseminate information about TTF. Simplified visual aids, culturally appropriate messaging, and targeted social media campaigns have been effective in raising awareness of novel cancer treatments. For example, a recent study found that social media campaigns targeting rural demographics increased awareness by 50% within three months [21]. Partnering with local organizations to deliver educational workshops or informational materials could also help address gaps in understanding and combat misinformation [22]. These strategies should be designed to meet the unique needs of rural populations, including varying literacy levels and cultural contexts.

Collaborations between academic institutions and rural healthcare providers could foster sustainable solutions to improve access to advanced treatments [23]. Partnerships focused on research, training, and resource sharing have successfully addressed healthcare disparities in other oncology fields by building local expertise. For example, academic institutions could provide training programs for rural healthcare providers on the implementation and management of TTF while also conducting community-based research to better understand barriers to adoption. Such collaborations could empower rural healthcare systems to support patients in adopting TTF and similar therapies, fostering long-term improvements in neuro-oncology care.

This study has several limitations, including its reliance on self-reported data, which could introduce biases such as overestimation of willingness to adopt TTF. Additionally, the sample may not fully represent all rural communities, given its focus on Michigan. Future research should explore a broader geographic scope to determine whether these findings hold across diverse rural populations and identify additional factors influencing TTF adoption. The absence of clinical outcome data also limits the ability to evaluate the real-world impact of TTF adoption in rural settings. Addressing these limitations in future studies could provide a more comprehensive understanding of the barriers and facilitators of TTF adoption.

By addressing these barriers and leveraging community-driven initiatives, telemedicine, and targeted educational campaigns, stakeholders can reduce disparities and improve access to innovative therapies like TTF. These efforts represent an essential step toward achieving equitable cancer care for rural populations, serving as a framework for addressing broader healthcare disparities in underserved communities.

## Conclusions

This study underscores the potential to enhance awareness and adoption of TTFs among rural populations through targeted, evidence-based interventions. Despite low baseline awareness of TTF (30; 25%), the high willingness to adopt the therapy when recommended by healthcare providers (86; 72%) highlights a critical opportunity to bridge knowledge gaps. Key barriers, including cost (78; 65%), geographic inaccessibility (66; 55%), and lack of understanding (60; 50%), underscore the need for comprehensive strategies to reduce disparities and expand access to advanced neuro-oncology treatments in underserved communities. Efforts to address these barriers should focus on implementing tailored interventions that combine educational, technological, and financial support. Specific strategies could include telemedicine expansion to mitigate geographic isolation, culturally tailored educational campaigns to improve health literacy, and financial assistance programs, such as subsidies or sliding-scale payment models, to alleviate cost-related challenges. Collaborations between academic institutions, rural healthcare providers, and community organizations will be essential to ensuring these solutions are sustainable and accessible.

While these findings provide valuable insights, this study has limitations that warrant further investigation. The reliance on self-reported data introduces potential biases, including social desirability effects, which may influence reported willingness to adopt TTF. Additionally, the cross-sectional design prevents the assessment of long-term changes in awareness and adoption. Future research should employ longitudinal designs to track shifts in TTF awareness and adoption over time, as well as explore additional rural regions to assess the generalizability of these findings. Including income-specific data and clinical outcome measures could provide a more comprehensive understanding of financial constraints and treatment efficacy. By prioritizing equity in neuro-oncology care, stakeholders can foster sustainable and impactful improvements in rural healthcare systems. Bridging the gap in TTF access represents a broader commitment to addressing healthcare disparities and improving outcomes for GBM patients in underserved areas.
